# The NeST (Nephrotic Syndrome Trust) App, a novel, co-designed self-management support app for young people and young adults with Nephrotic Syndrome: a multi-method survey reporting initial app development and evaluation

**DOI:** 10.1186/s12882-025-04684-1

**Published:** 2025-12-15

**Authors:** Moin A. Saleem, Wendy Cook, David Cook, Reihaneh Damghani, Maryam Afzal, Retha Steenkamp, Steve Donovan, Veronica Swallow

**Affiliations:** 1 University of Bristol, Bristol, UK; 2 Nephrotic Syndrome Trust (NeST), Salisbury, UK; 3 WEP Clinical, London, UK; 4 UK Renal Registry, Bristol, UK; 5 Citrus Suite Ltd, Liverpool, UK; 6 Sheffield Hallam University, Sheffield, UK

**Keywords:** Nephrotic Syndrome, Young people and young adults, NeST app, Multi-methods survey, Qualitative, Quantitative

## Abstract

**Background:**

There is a need for a user-led, evidence-based digital application (app.) that meets the identified information and support needs and preferences of young people and young adults aged 12–35 years (YP/YA) with Nephrotic Syndrome (NS) in the United Kingdom (UK). The password protected novel Nephrotic Syndrome Trust (NeST) app was therefore co-designed with YP/YA with NS to empower them to: access news of NS related events, take more control of their treatment and feel confident in sharing and accessing their data. The app allows YP/YA with NS to record regular urine dipstick readings, blood pressure, weight, temperature, medications, immunisations, symptoms (e.g. swollen feet), relapse or remission episodes, and the name of their renal unit. Additional features include an appointment diary to record feedback from their renal multidisciplinary team, treatment information and hospital admission episodes. The software was approved for release on iOS & Android app stores and the NHS Digital verification programme, meaning that users can be identified against their NHS records. The aim of the survey was to evaluate the NeST App from the perspective of YP/YA with NS.

**Methods:**

Through a consultative process, an online survey involving a combination of closed and open-ended questions was created and circulated via social media and email to target users of the app.

**Results:**

Twenty YP/YA with NS aged 12 years and older tested the app, completed the survey and provided quantitative and qualitative data. All found this app helpful, and easy to use and all would use it in future as part of standard practice.

**Conclusions:**

These data provide important feedback and suggestions for further app refinement and will integrate it with current national data collection via the UK Renal Registry (UKRR). To build on this collaborative project the developers will continue to collaborate with patients and health care professionals to ensure the app is a continually evolving and relevant resource, providing a voice for those living with NS. The app technology could potentially be rebooted and relaunched at minimal cost to support patients with other kidney conditions.

**Clinical trial number:**

Not applicable.

**Supplementary Information:**

The online version contains supplementary material available at 10.1186/s12882-025-04684-1.

## Background

Idiopathic Nephrotic Syndrome (NS) is a rare renal disease that can lead to significant morbidity, including kidney failure, transplant failure and in severe cases, mortality [1]. Young people and young adults (YP/YA) diagnosed with NS represent a particularly vulnerable subgroup of renal patients. This population often demonstrates limited engagement with traditional support networks, instead showing a preference for digital forms of communication and support. who tend not to engage in traditional patient support networks,

Evidence suggests that co-designed, evidence-based digital interventions can offer YP/YA accessible medical, educational, vocational, and psychosocial support not typically available through conventional care pathways.

However, recent literature reviews [2–4] have highlighted a notable paucity of such interventions, report a paucity of co-designed, evidence-based apps to support YP/YA living with long-term conditions such as NS. Qualitative studies exploring desirable components for customized, home-based, digital self-management resources for YP/YA with long-term conditions recommend that development of digital apps to meet YP/YAs’ identified information and support needs and preferences could enhance the apps’ uptake and utility, thereby augmenting self-management and optimizing clinical outcomes [5, 6]. This evidence-based approach to app design and development is in stark contrast to the thousands of apps available on the app market that are not evidence-based or user- or professional-informed. This dearth of suitable apps emphasises the need for studies of the development, evaluation, and effectiveness of mobile apps to support YP/YAs’ living with NS.

## The Nephrotic Syndrome Trust (NeST) App development

To address this identified gap, the NeST app was developed by a collaboration involving the Nephrotic Syndrome Trust (NeST), Citrus Suite Ltd., and RaDaR (the National Registry of Rare Kidney Diseases). RaDaR, the largest, rare kidney disease registry in the world, is a powerful source of real-world data that can inform understanding of rare kidney conditions and related research [7, 8]. The app was designed to empower YP/YA with NS in the United Kingdom (UK) by enabling them to access news of NS related events, take more control of their treatment and feel confident in sharing their data and experiences with healthcare professionals. The app allows patients to record regular urine dipstick readings, blood pressure, weight, temperature and symptoms that are prevalent with their condition e.g., swollen feet and ankles; in addition, any relapse or remission medication taken can be tracked. The app also records the name of the YP/YA’s renal unit, what concomitant medication is taken, and their immunisation status. Citrus Suite worked with NeST and the NeST Young Ambassadors group to design the app and enrich it with bespoke features that include an appointments diary which allows users to record Consultant feedback, treatment information and hospital admissions details. An additional file of screenshots from the app illustrates these features in more detail (see Additional file 1). The software received approval from both Apple and Google for distribution via the iOS and Android app stores. Additionally, its developer, Citrus Suite Ltd., was accepted into the National Health Service (NHS) Digital Verification Programme, enabling the application to verify users against their NHS records. The aim of the current survey was to evaluate the NeST App from the perspective of YP/YA with NS.

## Methods

### Evaluation design

Using a multi-method design and with support from a Young Ambassadors’ Group established and led by the charity NeST, we developed a set of closed and open-ended survey questions, a participant information sheet and invitation text (Appendices 1 & 2) to enable us to meet our objective. After several iterations of the questions, we piloted the survey with a healthy young person aged 12 years of age and made the suggested revisions to help ensure its appropriateness for young people.

### Setting, respondents and data collection

The final survey questions, with a combination of ‘yes or no’ and free text answers (Appendix 2) were entered into Google Forms https://surveys.google.com/your-surveys and a link to the survey along with an invitation and Participant Information Sheet (Appendix 1) were distributed to YP/YA living with NS via email, Twitter (now rebranded to X) and the closed NeST Young Ambassadors Facebook group. Quantitative and qualitative data were collected.

**Fig. 1:**
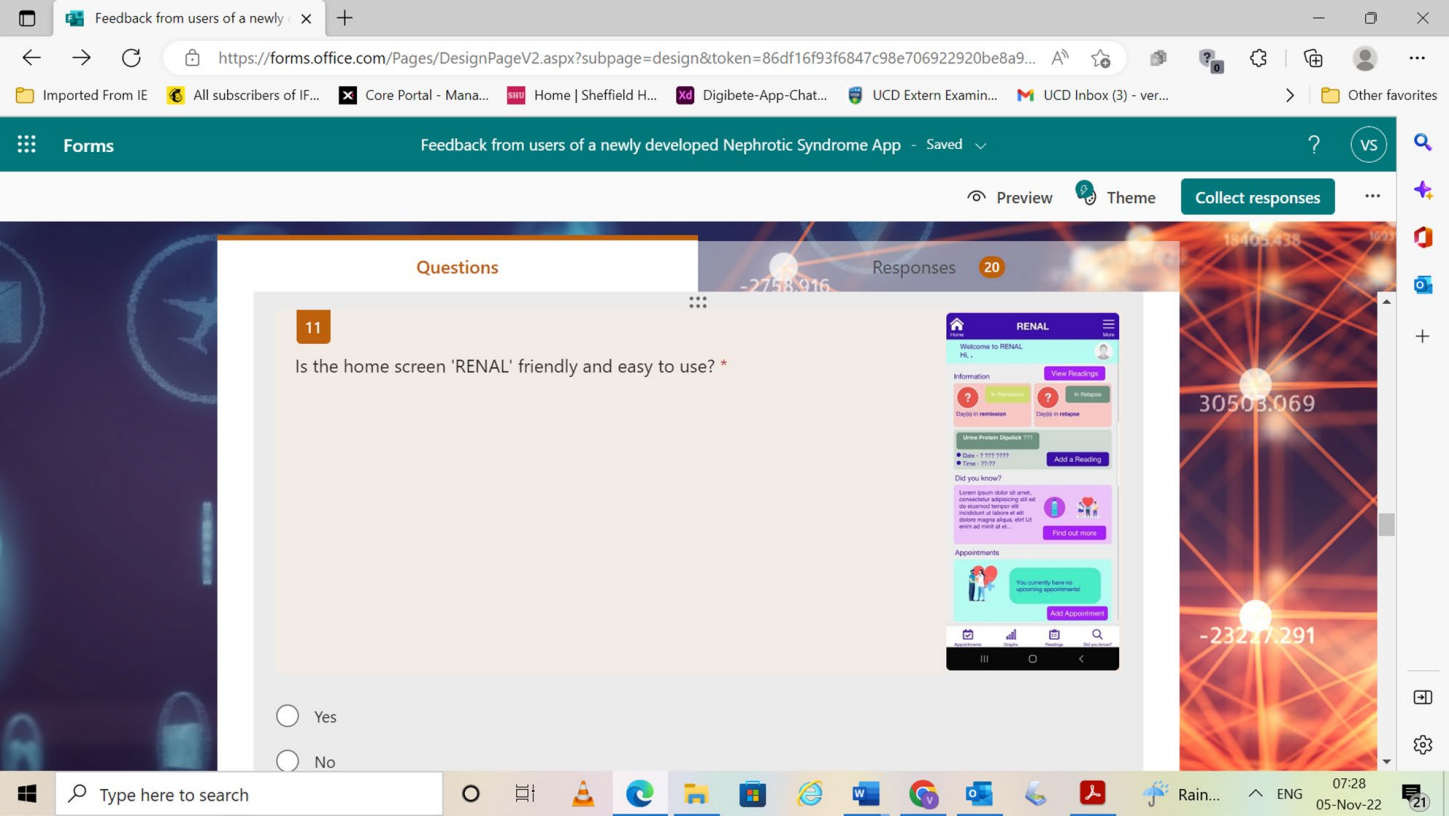
Example of a question in the survey

Figure 1 illustrates an example question as it appeared to respondents on their digital device or PC screen:

### Data analysis

#### Statistical analysis

Quantitative data were analysed within Google Forms. Descriptive statistics were used to summarise respondents’ characteristics. Categorical data were summarised using frequencies and percentages, and quantitative data summarised using means (SD) for normally distributed data or median range for ordinal data.

#### Analysis of qualitative free-text comments

Qualitative data were analysed using thematic analysis, a method well-suited to this study as it facilitates both inductive and deductive identification of themes and patterns and ensures trustworthiness and reliability. Two experienced qualitative researchers conducted the analysis, drawing upon their extensive theoretical and conceptual understanding of app development and evaluation, while remaining open to emergent insights from the data [9, 10].

The analysis followed a five-stage process designed to enhance trustworthiness and reliability:

1. **Familiarisation**: Both analysts independently reviewed the entire dataset and conducted initial coding on a sample of transcripts.
2. **Preliminary Coding Consensus**: The analysts compared and discussed their individual coding to reach agreement on preliminary coding concepts.
3. **Development of Coding Framework**: Six thematic domains were identified and used to construct a coding framework, which was then systematically applied across the full dataset.
4. **Interpretative Validation**: Data excerpts were shared to stimulate further discussion, refine interpretations, and ensure alignment with the study’s aims. Analysts referred back to the raw data as needed to clarify meaning and ensure analytic depth.
5. **Reporting**: The final analysis report was produced, incorporating illustrative verbatim quotations from respondents. These were contextualised within the thematic discussion and visually supported by Word Clouds [11–14], examples of which are presented in Additional File 2 .

## Survey results

### Numerical results

Responses were received from 20 out of 22 = 91% completion rate (two of the initial respondents did not complete the survey as their health had deteriorated after initially agreeing to participate and they were in hospital). The ’ average time to complete the survey was 04:04 min. Results obtained are presented below in Table 1.

**Table 1:** Results for fixed-choice survey questions (*n* = 20; completion rate = 91%)

Category	Survey Question (Q#)	Variable / Parameter	Response (%)	No. of Respondents	Interpretation / Comment
**Demographics**	Q3	Female	55%	11	Majority were female
		Male	45%	9	
	Q2	English as first language	95%	19	High English proficiency among
	Q1	12–15 years old	35%	7	Largest age subgroup
		16–19 years old	10%	2	
		20–25 years old	5%	1	
		26–30 years old	15%	3	
		31–35 years old	20%	4	
		No response	15%	3	
**Condition management**	Q5	Receive help from parent/guardian	50%	10	Half required caregiver support
**Symptom recognition**	Q6	Noticed symptoms after > 5 years	50%	10	Delayed recognition common
		Noticed symptoms in 3–5 years	20%	4	
		Noticed symptoms within 12 months	30%	6	Early recognition less frequent
**App usage**	Q4	Do not use other Apps/devices	80%	16	Potential novelty of App use in this group
**Perceived usefulness**	Q7	Found the App helpful	100%	20	Universal positive response
**Ease of use**	Q9	Found the App easy to use	100%	20	Strong usability
**Support needs**	Q10	Would need help using the App	15%	3	Minority required assistance
**Willingness to use**	Q8	Would use the App	100%	20	Complete acceptance
**Interface evaluation**	Q11	RENAL homepage friendly/easy	95%	19	High satisfaction
	Q13	ADD APPOINTMENT Page friendly/easy	100%	20	Full satisfaction
	Q15	GRAPHS Page friendly/easy	100%	20	
	Q17	ADD A READING Page friendly/easy	100%	20	
	Q19	DID YOU KNOW Page friendly/easy	100%	20	

### Qualitative findings

Six survey questions (12, 14, 16, 18, 20 and 21) offered an opportunity for respondents to provide additional free text answers. Analysis of the free-text qualitative comments led to the identification of six themes; these are described and discussed below, and typical verbatim quotations from within these themes are cited in Additional File 3 [9]. In addition, word clouds (as displayed in Additional File 2) were used for visualizing unstructured text data and obtaining insight into trends and patterns within each of the themes/sub-themes.

#### Theme 1- Improving the RENAL Screen?

Seven respondents said they did not want any improvements made to the app as they found it very helpful in enabling them to keep track of their NS management. However, some quotations provided suggestions for improvement in the following areas:

##### Medication

Two respondents suggested the addition of a section in the app on medication, and one of these specifically requested an option for low dose prednisolone.

##### Fluid restriction and dipsticks

Concern was expressed about the lack of a facility for documenting fluid restrictions, self-monitoring fluid intake and recording signs of oedema, because these are such important aspects of NS self-management. In addition, one young person would like the app to provide support with interpreting the results in a urine dipstick test.

##### Relapse and remission

some comments related to a desire for a process for interpreting the results in a urine dipstick, and *one respondent recommended a facility to enable them to select ‘remission’ or ‘relapse’ and if relevent*,* to enter how many days they had been in remission.*

##### Accessibility and appearance

Although most young people were happy with the appearance of the app and found it easy to access and use, two respondents made suggestions for improving the use of colours on the screen, feeling that some of the colours seemed more likely to appeal to people who were visually impaired.

#### Theme 2: Improving the ADD APPOINTMENT screen

Fifteen young people responded to this question, and most were very happy with this feature, as can be seen from the word cloud in Additional File 2.

However, four improvement suggestions were made regarding adding the location of the appointment or whether it was a telephone appointment, with a search button for the hospital and location; help to set a reminder; and a recurring appointment or ‘on-going’ treatment option for outpatients’ appointments.

#### Theme 3: Improvement of the GRAPHS screen

Most respondents were happy or very happy with this feature and would not change anything, as they enjoyed the opportunity to be able to view the progress of their NS. Nevertheless, two respondents made helpful suggestions for improving the GRAPHS screen including (i) being able to view their data from the previous year and (ii) the addition of an albumin results section as patients with NS may find it helpful to see what their albumin levels are as they understand that it can be an indicator of whether their NS is improving or deteriorating.

#### Theme 4: Improving the ADD A READING screen

Of the fourteen responses in this theme, three provided useful suggestions for enhancing this section of the app. These included (i) adding an extra ‘symptom notes box’ for symptoms that are not linked to oedema or temperature; and (ii) making it possible for a YP to select more than one option in the oedema section by presenting it as a list rather than a drop-down option and, (iii) providing an option to copy the previous test result such as protein tests instead of having to fill out all of the details for the same result every day.

#### Theme 5: Improving the DID YOU KNOW screen

Of the eleven responses received, eight were happy with this screen, saying for example that it was very informative and that they really appreciated the information therein. Suggestions for improving this screen included adding under each section general advice for that topic. Finally, one young person asked for reassurance that the information contained in the app would be updated regularly.

#### Theme 6: Further comments

Some further constructive comments were offered, for example there were requests for the instructions on the process for adding health data to be simplified and a more streamlined editing process so that typographical errors made by users when entering their data could be easily edited. Another request was for a peer -to -peer support forum on the app so that YP could communicate with other YP with NS. The final suggestion was for inclusion of advice on light exercise that would be suitable for YP with NS.

## Discussion

This project aimed to survey, using email and social media, a UK sample of YP/YA living with the rare disease NS. Social media as a recruitment and data collection method is increasingly used by researchers; advantages of this approach include speed of recruitment, cost-efficiency, snowballing effects, and accessibility of the researcher to potential respondents. The use of email and social media to recruit study respondents is therefore, a feasible, inexpensive, and efficient approach to recruiting a diverse sample for survey research [10–12]. The collection of valid and reliable survey data, including from YP/YAs is of great importance in healthcare. However, numerous methodological and practical problems arise in the planning and collection of such survey data that need to be resolved to maximize the response rate without being prohibitively costly and to ensure the validity of the data collected.

We received anonymised responses from 20 respondents aged over 12 years old, the majority were aged between 12 and 35 years although 15% of the sample did not state their age. Through a combination of closed and open-ended questions we obtained some rich and valuable data from YP/YA living with NS; these data provide important feedback and suggestions for further refinement of the app before it is implemented into standard care. The main message from this survey is that all found this App helpful, and easy to use and all would use it in future if it became part of standard practice. In addition, all found the ‘ADD APPOINTMENT’, ‘GRAPHS’, ‘ADD A READING’ and ‘DID YOU KNOW?’ pages friendly and easy to use, while 95% found the ‘RENAL HOMEPAGE’ friendly and easy to use. 80% of said they do not use any other App or device to manage their NS and many stated that they would very much enjoy using this app in the future, although of the total respondents, 15% reported they would need help to use the App. An interesting observation is that in response to Q6, 50% of respondents said it took 5 years or more to notice the symptoms of NS, this is a well-recognised problem that is associated with the lack of early recognition of this condition. 80% of respondents said they do not use any current device or app to help self-manage their NS. It is unclear, however, why they do not for example use the current Patients Know Best platform https://patientsknowbest.com/ or any other relevant app or platform; this may be because they are unaware of their availability or of their potential benefit for patients. As recommended in the literature, these findings confirm the importance of involving YP/YA [2, 5, 13, 14] in the co-design, development and evaluation of digital self-management resources such as this app. This is more likely to result in user-friendly and developmentally appropriate digital resources that meet the needs of the target population. A summary of the key recommendations for refinement arising from the survey is provided in Appendix 3.

### Strengths, limitations, and recommendations

A strength of this project is that the technology behind the app can be rebooted and relaunched to support patients with other kidney conditions at minimal cost. The survey results are being used to make improvements in the app. In addition, the survey methods used made this approach valuable in the context of a pandemic where families were less likely to attend healthcare appointments in person and provides a robust system for self-monitoring between appointments. The multi-method research design involving a combination of open ended and closed questions has resulted in rich data to inform future app developments. In addition, these data provide important feedback and suggestions for further app refinement before it is implemented into standard care and will integrate with current national data collection via UKRR, so will form part of the collective research outputs from the large cohorts represented.

We believe the sample is likely to be representative of YP/YA with NS for two main reasons: (i) the app was piloted by members of our advisory group—including a young adult with NS and a healthy 12-year-old, and both found it accessible and user-friendly, reflecting the general digital literacy of our target age group in the UK; and (ii) the survey was distributed via email and a closed Facebook group for Young NS Ambassadors, these platforms (email and Facebook) are widely used by YP/YA in the general population. These factors suggest our sample reflects the typical communication and technology engagement behaviours of the wider NS population. The app was approved for release on iOS & Android app stores and the developer, Citrus Suite, were accepted onto the NHS Digital verification programme; the app has now been live for 18 months. Any coding and issues with the app can be addressed in the next update, if required.

Based on the design and methodology we developed and used in this multi-method survey and as reported here, the technology behind the app can in the future be rebooted and relaunched to support patients with other kidney conditions at minimal cost.

Limitations include the small sample size and some missing data but as NS is a rare disease [1, 15], and the project was undertaken against a backdrop of the Covid-19 pandemic [16] when the rise in survey distribution on social media was found to result in survey fatigue in the wider population, reduced response rates and variable data quality amongst patients [10] is not surprising. In addition, we did not include a facility for peer-to-peer interaction by app users, but in response to user feedback the intention is for a future iteration of the app to be co-designed to safely facilitate peer to peer interaction. In addition, we did not formally assess whether respondents were particularly tech-savvy or highly motivated and therefore cannot directly compare them to the broader NS population. These factors may help explain why our sample size was small. In addition, our survey design meant we were unable to explore in more depth respondents’ responses to questions, and we did not collect data on long-term usage or sustainability.

Future qualitative research involving semi-structured interviews could explore these issues in more depth, for example it could investigate patients’ awareness of and views on existing apps and platforms such as the Patient Knows Best platform https://patientsknowbest.com/. Currently, only people registered on RaDaR can access the NeST app. Future NeST workshops will be held at six -monthly intervals to re-evaluate the app and help to ensure it is reaching its full optimal effectiveness for the users. Optimising this patient held resource aims for improving understanding of disease at the patient level and working towards utility for broader research questions and clinical trials.

## Conclusion

This survey has, through using a combination of closed and open-ended questions, obtained the views of YP/YAs living with NS on a purpose designed app to support self-management. The findings provide new information to support further app refinement and its future implementation into standard practice and inform ongoing development of the app. It is important for renal multi-disciplinary teams, researchers and patients and their families to sustain this collaboration to ensure that the app is a continually evolving resource, providing a voice for those living with NS and to ensure that the app does not become redundant.

## Supplementary Information

Below is the link to the electronic supplementary material.

Supplementary Material 1

Supplementary Material 2

Supplementary Material 3

Supplementary Material 4

## Data Availability

Available upon request from corresponding author.

## Abbreviations

CKD: Chronic kidney disease
NS: Nephrotic Syndrome
NeST: Nephrotic Syndrome Trust
RaDaR: The National Registry of Rare Kidney Diseases (RaDaR) is a Renal Association initiative designed to pull together information from patients with certain rare kidney diseases

## References

[1] Saleem M, Pitcher D, Barratt J, Braddon F, Gong W, Hendry B, et al. Natural history of idiopathic nephrotic syndrome: the UK National RADAR idiopathic nephrotic syndrome cohort. Nephrol Dialysis Transplant. 2024;39(Suppl_1):#901.

[2] Edwards J, Waite-Jones J, Schwarz T, Swallow V. Digital technologies for children and parents sharing self-management in childhood chronic or long-term conditions: A scoping review. Children. 2021;8(12):1203. 34943399 10.3390/children8121203 PMC8700031

[3] Majeed-Ariss R, Baildam E, Campbell M, Chieng A, Fallon D, Hall A, et al. Apps and adolescents: A systematic review of adolescents’ use of mobile phone and tablet apps that support personal management of their chronic or Long-Term physical conditions. J Med Internet Res. 2015;17(12):e287. 26701961 10.2196/jmir.5043 PMC4704897

[4] Nightingale R, McHugh G, Kirk S, Swallow V. Supporting children and young people to assume responsibility from their parents for the self-management of their long-term condition: an integrative review. Child Care Health Dev. 2019;45(2):175–88. 30690751 10.1111/cch.12645

[5] Nightingale R, Hall A, Gelder C, Friedl S, Brennan E, Swallow V. Desirable components for a Customized, Home-Based, digital Care-Management app for children and young people with Long-Term, chronic conditions: A qualitative exploration. J Med Internet Res. 2017;19(7):e235. 28676470 10.2196/jmir.7760 PMC5516103

[6] Swallow VM, Hall AG, Carolan I, Santacroce S, Webb NJ, Smith T, et al. Designing a web-application to support home-based care of childhood CKD stages 3–5: qualitative study of family and professional preferences. BMC Nephrol. 2014;15:34. 24548640 10.1186/1471-2369-15-34 PMC3933188

[7] Colby E, Hayward S, Benavente M, Robertson F, Bierzynska A, Osborne A, et al. National unified renal translational research enterprise: idiopathic nephrotic syndrome (NURTuRE-INS) study. Clin Kidney J. 2024;17(8):sfae096. 39135942 10.1093/ckj/sfae096 PMC11317841

[8] Taal MW, Lucas B, Roderick P, Cockwell P, Wheeler DC, Saleem MA, et al. Associations with age and glomerular filtration rate in a referred population with chronic kidney disease: methods and baseline data from a UK multicentre cohort study (NURTuRE-CKD). Nephrol Dial Transpl. 2023;38(11):2617–26. 10.1093/ndt/gfad110 PMC10615633 37230953

[9] Nowell LS, Norris JM, White DE, Moules NJ. Thematic analysis: striving to Meet the trustworthiness criteria. Int J Qualitative Methods. 2017;16(1):1609406917733847.

[10] de Koning R, Egiz A, Kotecha J, Ciuculete AC, Ooi SZY, Bankole NDA, et al. Survey fatigue during the COVID-19 pandemic: an analysis of neurosurgery survey response rates. Front Surg. 2021;8:690680. 34458314 10.3389/fsurg.2021.690680 PMC8388838

[11] Kristjansson AL, Sigfusson J, Sigfusdottir ID, Allegrante JP. Data collection procedures for school-based surveys among adolescents: the youth in Europe study. J Sch Health. 2013;83(9):662–7. 23879786 10.1111/josh.12079

[12] Sharma A, Minh Duc NT, Luu Lam Thang T, Nam NH, Ng SJ, Abbas KS, et al. A Consensus-Based checklist for reporting of survey studies (CROSS). J Gen Intern Med. 2021;36(10):3179–87. 33886027 10.1007/s11606-021-06737-1 PMC8481359

[13] Ahmed SK. The pillars of trustworthiness in qualitative research. J Med Surg Public Health. 2024;2:100051.

[14] Majeed-Ariss R, Hall AG, McDonagh J, Fallon D, Swallow V. Mobile phone and tablet apps to support young people’s management of their physical Long-Term conditions: A systematic review protocol. Jmir Res Protoc. 2015;4(2):e40. 25854293 10.2196/resprot.4159 PMC4405621

[15] Vivarelli M, Gibson K, Sinha A, Boyer O. Childhood nephrotic syndrome. Lancet. 2023;402(10404):809–24. 37659779 10.1016/S0140-6736(23)01051-6

[16] Tse Y, Darlington AE, Tyerman K, Wallace D, Pankhurst T, Chantziara S, et al. COVID-19: experiences of lockdown and support needs in children and young adults with kidney conditions. Pediatr Nephrol. 2021;36(9):2797–810. 33742247 10.1007/s00467-021-05041-8 PMC7979448

