# Supplementary material for: The NeST (Nephrotic Syndrome Trust) App, a novel, co-designed self-management support app for young people and young adults with Nephrotic Syndrome: a multi-method survey reporting initial app development and evaluation
Source: BMC Nephrol. 2025 Dec 15;27:52. doi: 10.1186/s12882-025-04684-1 (PMC12822111; doi:10.1186/s12882-025-04684-1)
Supplement: Supplementary file 1 — Supplementary Material 1 [file 12882_2025_4684_MOESM1_ESM.pdf]

Additional File 1 _ Figures 1-6 of screen shots from the NeST App.

Fig_1

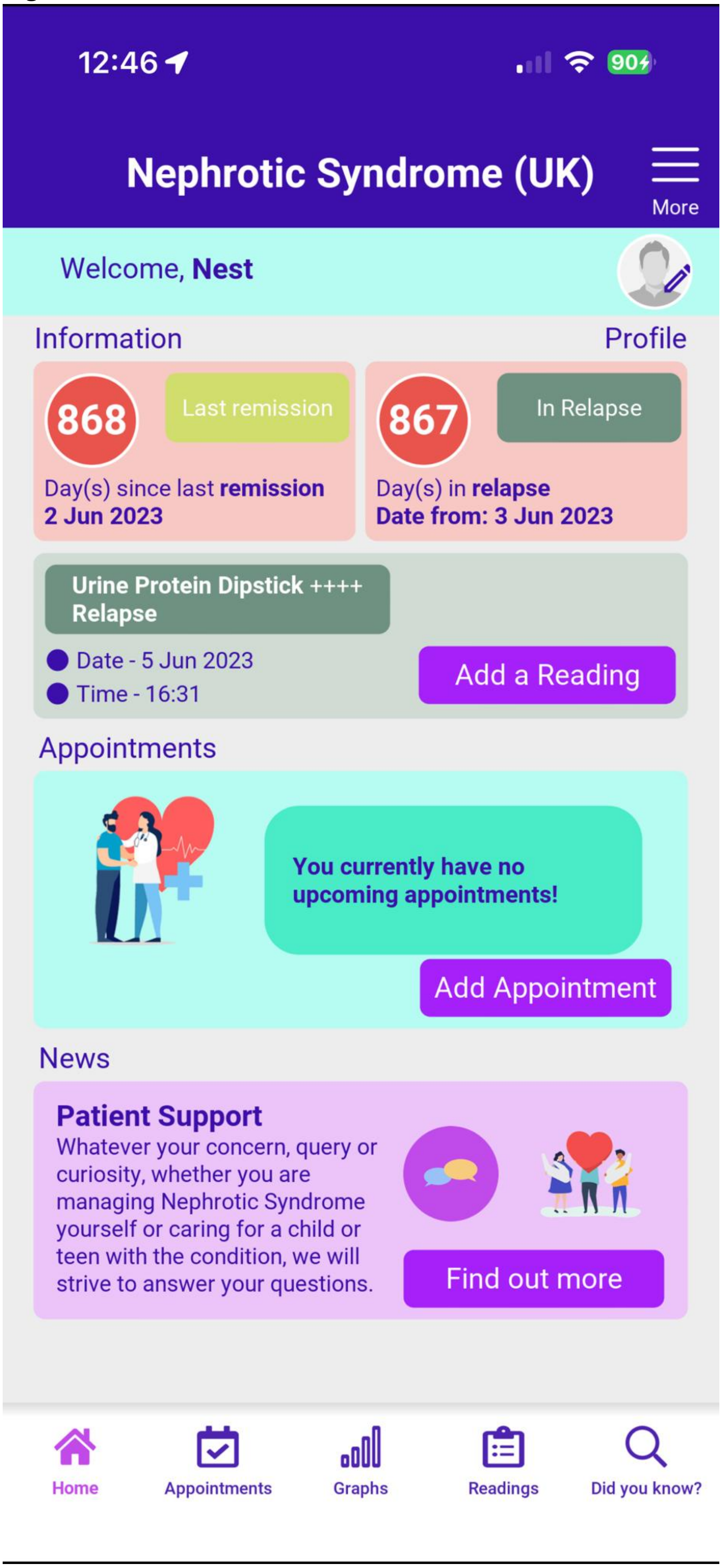

Fig_2

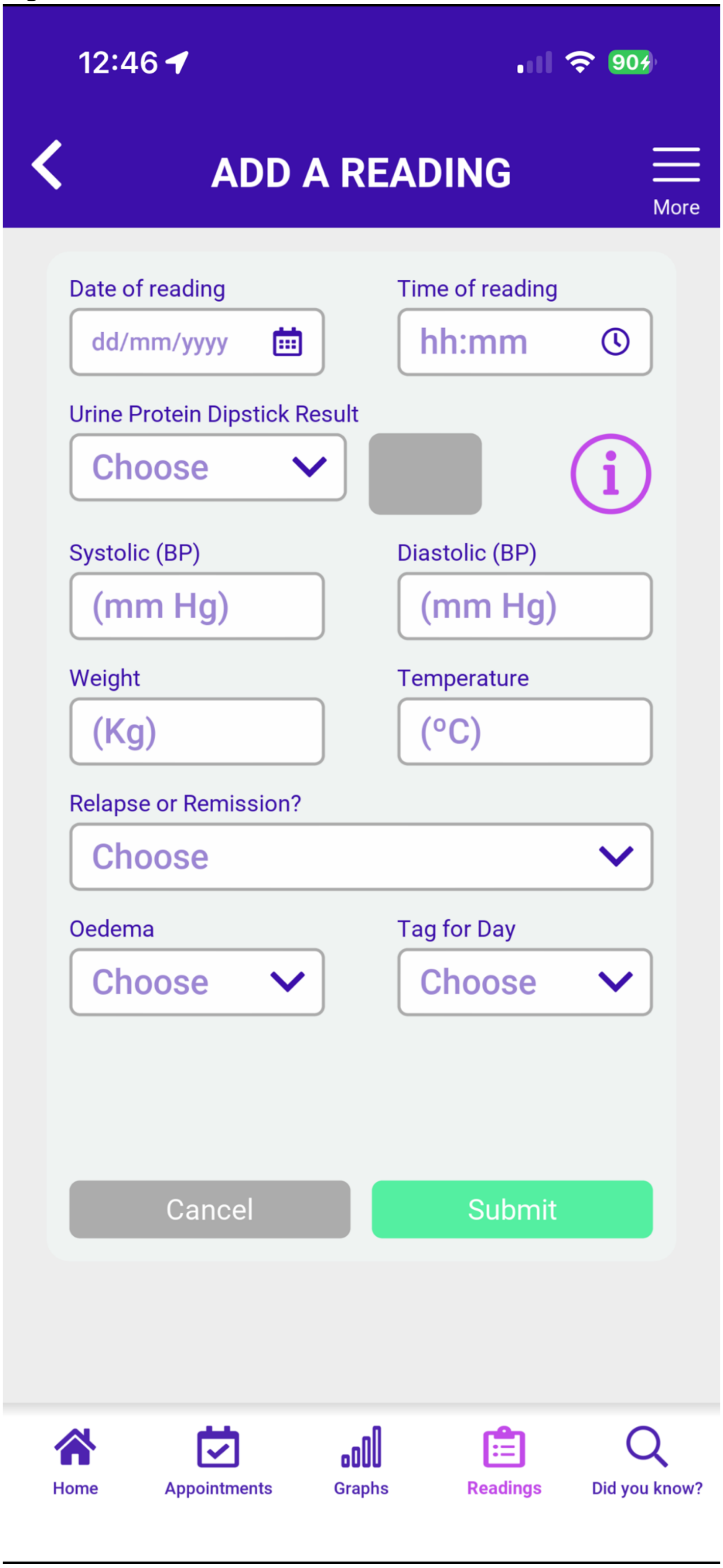
12:46
90
ADD A READING
More
Date of reading
dd/mm/yyyy
Time of reading
hh:mm
Urine Protein Dipstick Result
Choose
Systolic (BP)
(mm Hg)
Diastolic (BP)
(mm Hg)
Weight
(Kg)
Temperature
(°C)
Relapse or Remission?
Choose
Oedema
Choose
Tag for Day
Choose
Cancel
Submit
Home
Appointments
Graphs
Readings
Did you know?

Fig_2_1

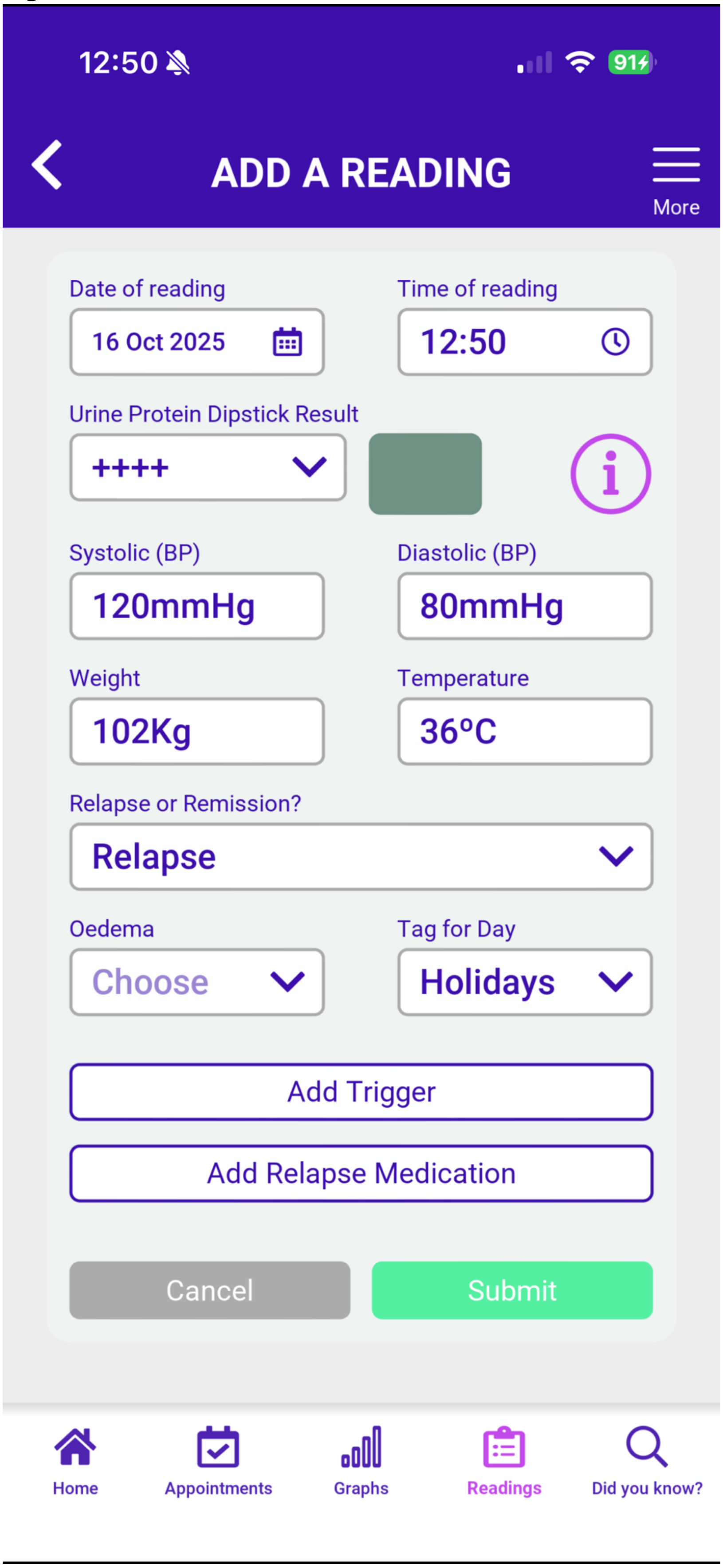
12:50
ADD A READING
More
Date of reading
16 Oct 2025
Time of reading
12:50
Urine Protein Dipstick Result
++++
Systolic (BP)
120mmHg
Diastolic (BP)
80mmHg
Weight
102Kg
Temperature
36°C
Relapse or Remission?
Relapse
Oedema
Choose
Tag for Day
Holidays
Add Trigger
Add Relapse Medication
Cancel
Submit
Home
Appointments
Graphs
Readings
Did you know?

Fig_2_2

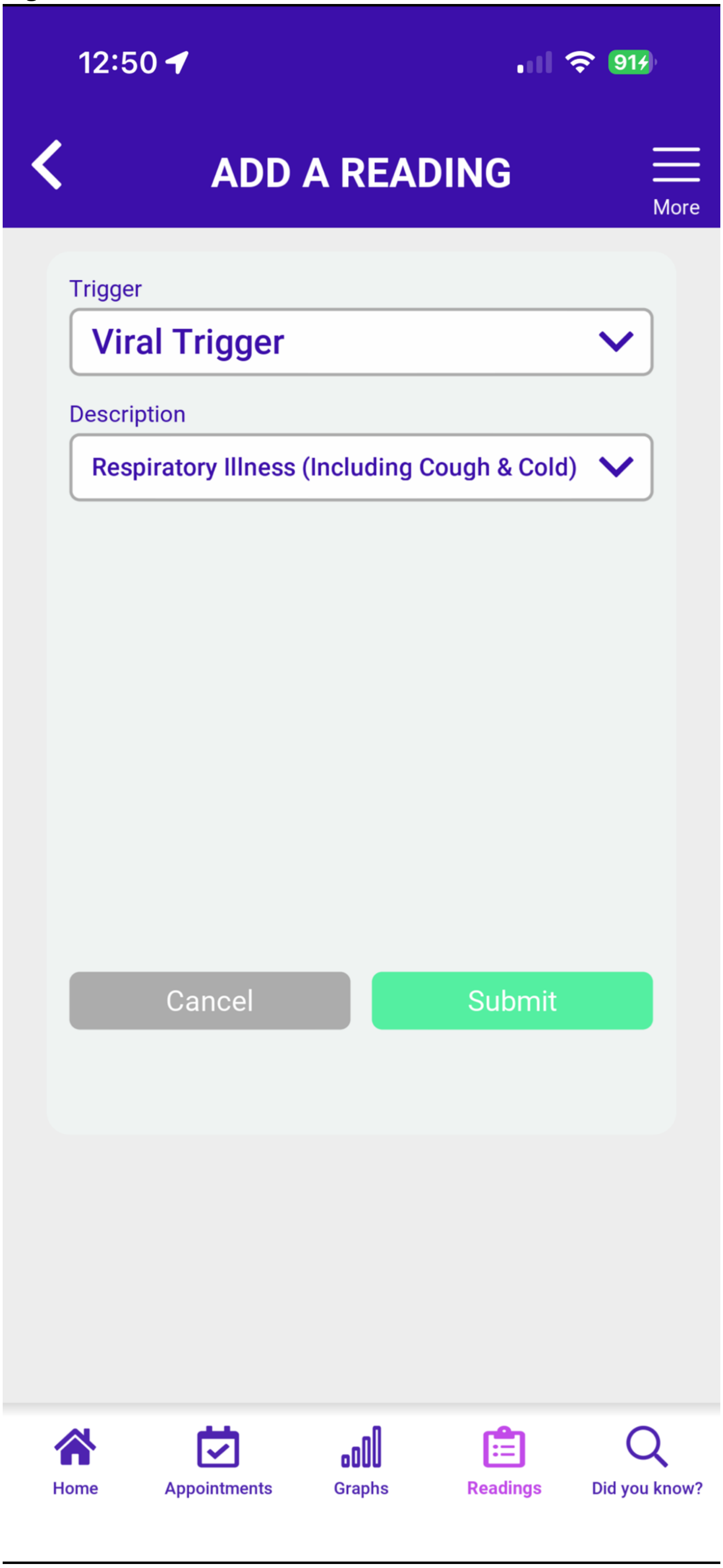
12:50
ADD A READING
More
Trigger
Viral Trigger
Description
Respiratory Illness (Including Cough & Cold)
Cancel
Submit
Home
Appointments
Graphs
Readings
Did you know?

Fig_2_3

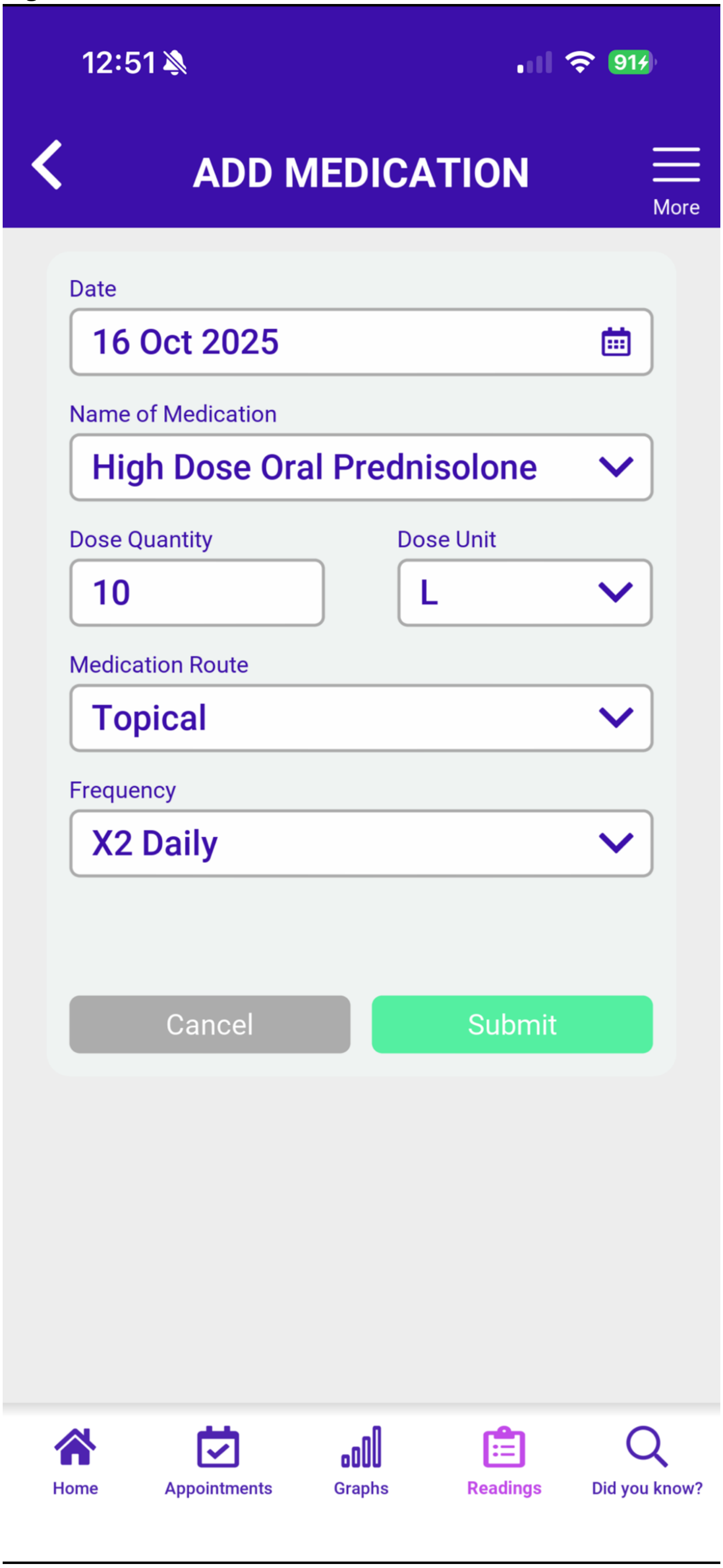
12:51
91
ADD MEDICATION
More
Date
16 Oct 2025
Name of Medication
High Dose Oral Prednisolone
Dose Quantity
10
Dose Unit
L
Medication Route
Topical
Frequency
X2 Daily
Cancel
Submit
Home
Appointments
Graphs
Readings
Did you know?

Fig_3

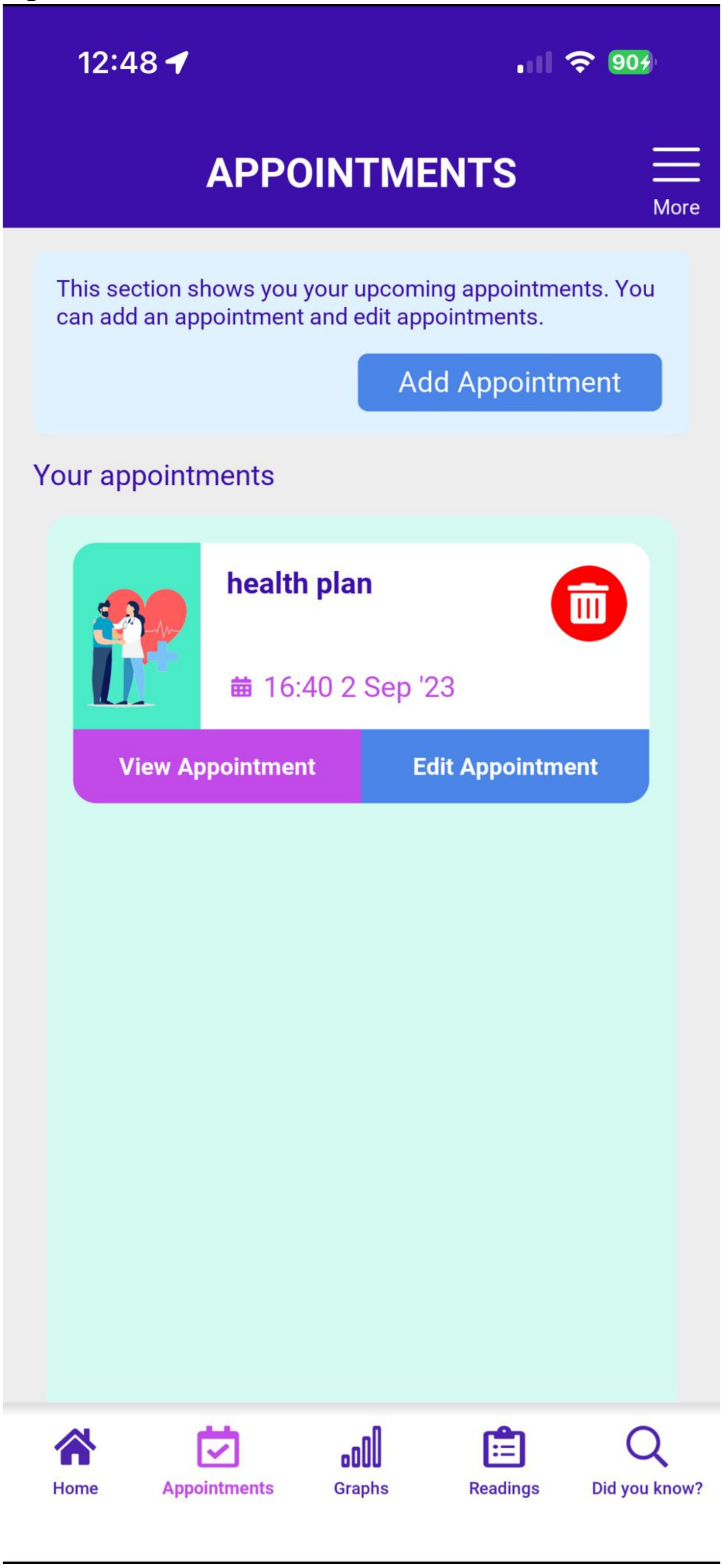
12:48
90
APPOINTMENTS
More
This section shows you your upcoming appointments. You can add an appointment and edit appointments.
Add Appointment
Your appointments
health plan
16:40 2 Sep '23
View Appointment
Edit Appointment
Home
Appointments
Graphs
Readings
Did you know?

Fig_4

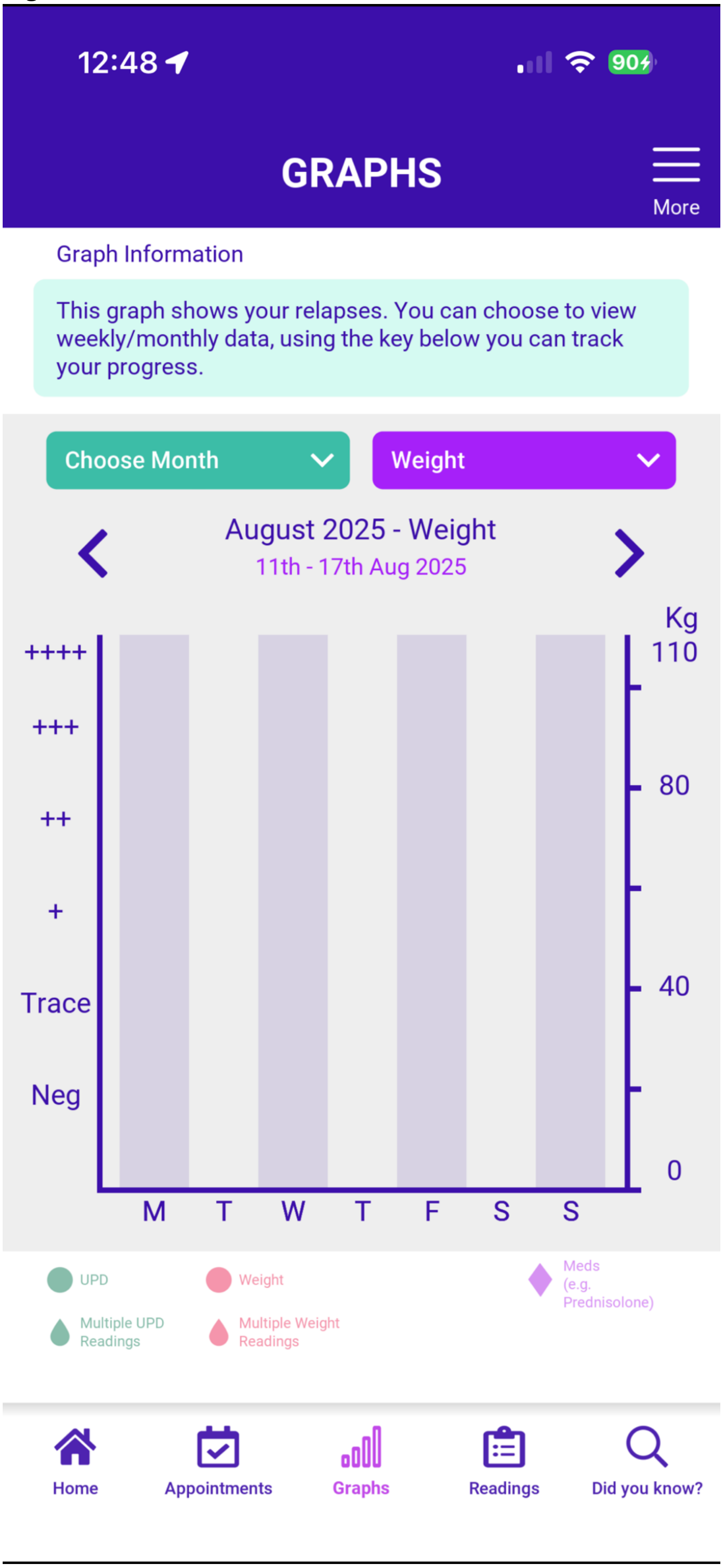

12:48
90
GRAPHS
More
Graph Information
This graph shows your relapses. You can choose to view weekly/monthly data, using the key below you can track your progress.
Choose Month
Weight
August 2025 - Weight
11th - 17th Aug 2025
Kg
110
80
40
0
++++
+++
++
+
Trace
Neg
M T W T F S S
UPD
Weight
Meds (e.g. Prednisolone)
Multiple UPD Readings
Multiple Weight Readings
Home
Appointments
Graphs
Readings
Did you know?

Fig_5

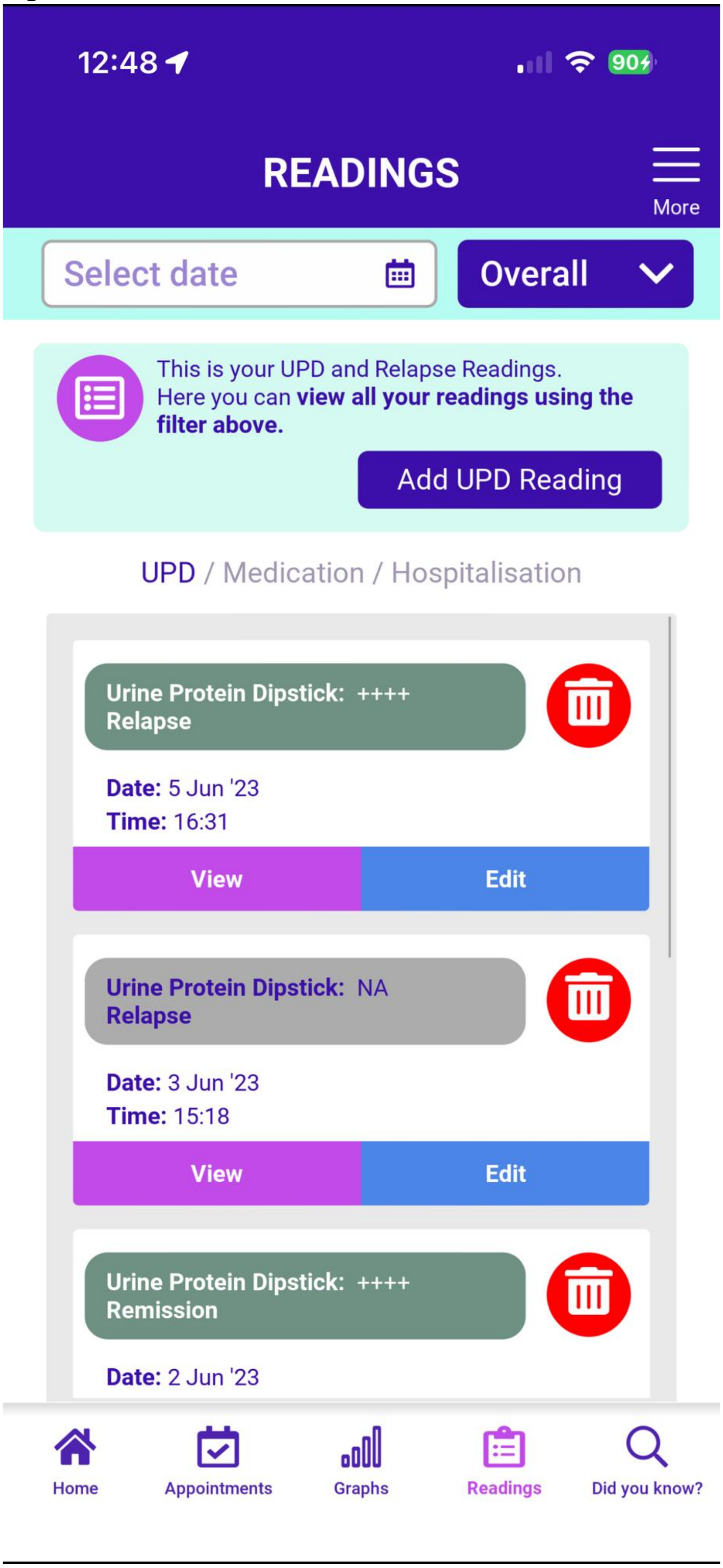
12:48
READINGS
More
Select date
Overall
This is your UPD and Relapse Readings.
Here you can view all your readings using the filter above.
Add UPD Reading
UPD / Medication / Hospitalisation
Urine Protein Dipstick: ++++
Relapse
Date: 5 Jun '23
Time: 16:31
View
Edit
Urine Protein Dipstick: NA
Relapse
Date: 3 Jun '23
Time: 15:18
View
Edit
Urine Protein Dipstick: ++++
Remission
Date: 2 Jun '23
Home
Appointments
Graphs
Readings
Did you know?

Fig_5_1

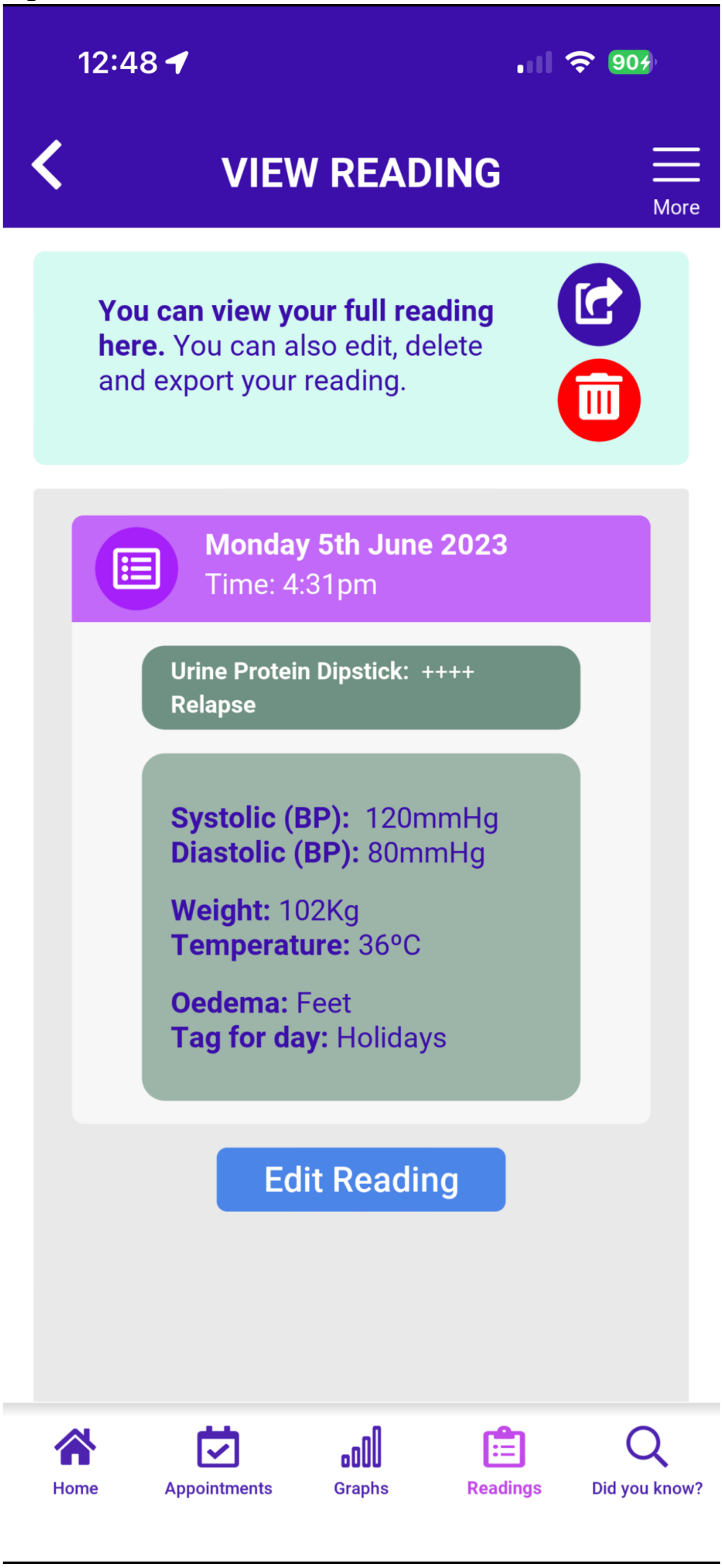

12:48
90
VIEW READING
More
You can view your full reading here. You can also edit, delete and export your reading.
Monday 5th June 2023
Time: 4:31pm
Urine Protein Dipstick: ++++
Relapse
Systolic (BP): 120mmHg
Diastolic (BP): 80mmHg
Weight: 102Kg
Temperature: 36°C
Oedema: Feet
Tag for day: Holidays
Edit Reading
Home
Appointments
Graphs
Readings
Did you know?

Fig_6

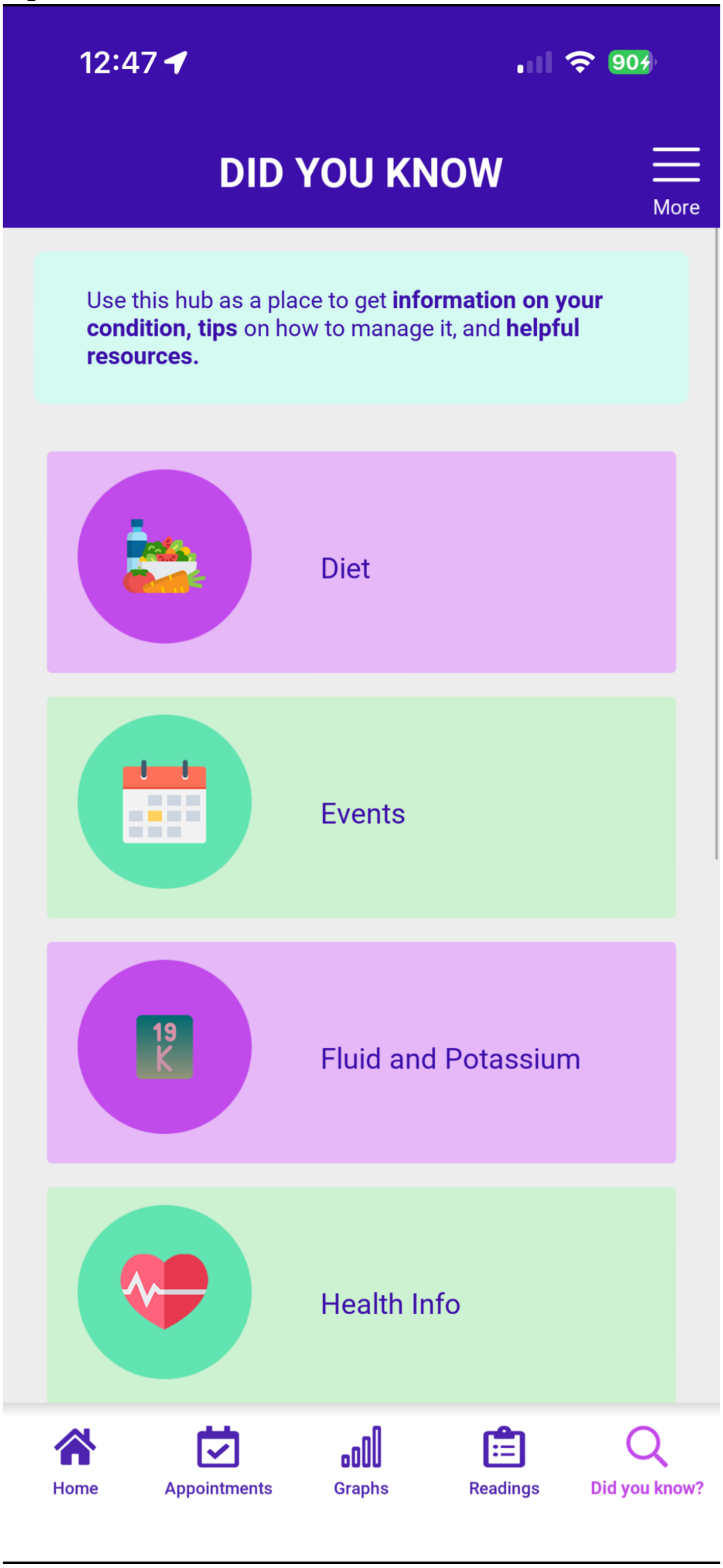
12:47
90
DID YOU KNOW
More
Use this hub as a place to get information on your condition, tips on how to manage it, and helpful resources.
Diet
Events
19
K
Fluid and Potassium
Health Info
Home
Appointments
Graphs
Readings
Did you know?

Fig_6_1

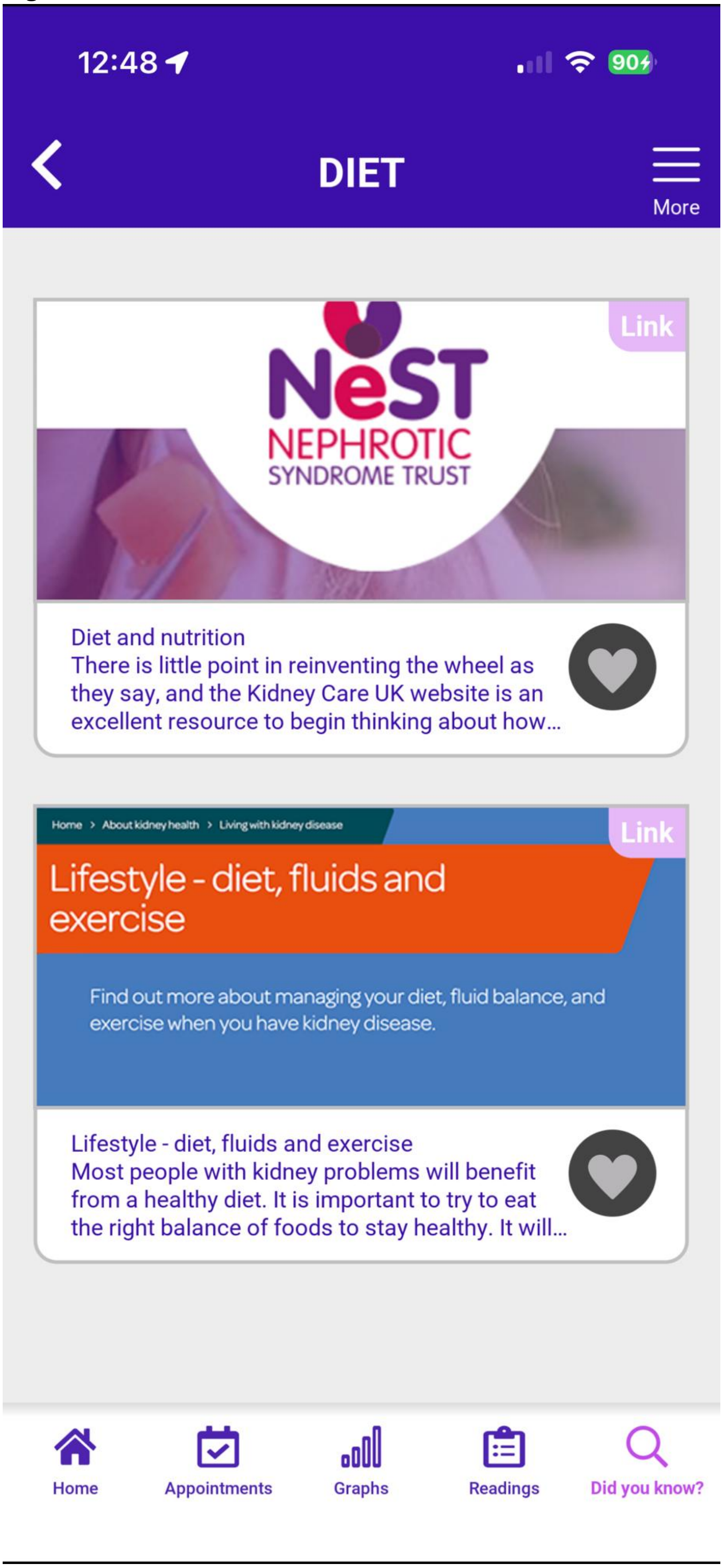
12:48
90
DIET
More
NeST
NEPHROTIC
SYNDROME TRUST
Link
Diet and nutrition
There is little point in reinventing the wheel as they say, and the Kidney Care UK website is an excellent resource to begin thinking about how...
Home > About kidney health > Living with kidney disease
Link
Lifestyle - diet, fluids and exercise
Find out more about managing your diet, fluid balance, and exercise when you have kidney disease.
Lifestyle - diet, fluids and exercise
Most people with kidney problems will benefit from a healthy diet. It is important to try to eat the right balance of foods to stay healthy. It will...
Home
Appointments
Graphs
Readings
Did you know?
