# Supplementary material for: The NeST (Nephrotic Syndrome Trust) App, a novel, co-designed self-management support app for young people and young adults with Nephrotic Syndrome: a multi-method survey reporting initial app development and evaluation
Source: BMC Nephrol. 2025 Dec 15;27:52. doi: 10.1186/s12882-025-04684-1 (PMC12822111; doi:10.1186/s12882-025-04684-1)
Supplement: Supplementary file 2 — Supplementary Material 2 [file 12882_2025_4684_MOESM2_ESM.docx]

## Additional file 3 : Verbatim quotations from survey respondents, organised by emergent themes/sub-themes

| **Themes** | **Subthemes** | **Verbatim quotations** |
| --- | --- | --- |
| ***Theme 1- Improving the RENAL Screen?*** | Medication  Fluid restriction and dipsticks:  *Relapse and remission:*  *Accessibility and appearance:* | *“I don’t think I would change anything it’s very well designed, and I find it rather helpful to keep track of things”*  *“I couldn't find an option for low dose prednisolone.”*  *“…to be able to add fluid restriction and monitor intake, would also be useful when looking at / comparing with oedema. Fluid restriction is really important when nephrotic!”*  *“Maybe explaining what the different things on the dipstick means”…*  *“I was unable to select 'remission' or 'relapse' to enter how many days I'd been in remission.”*  *“…[to be able to] type in a previous date to when you started remission rather than just when you started using the app”*  *“I feel the colours are a bit too much. Maybe that’s just personal preference, it doesn’t bother me or discourage from using the app though.*  *“Some of the colours seem a bit funky, but maybe that's for visually impaired people?”* |
| ***Theme 2: Improving the ADD APPOINTMENT screen*** |  | *“…adding the location of the appointment* [e.g. hospital or GP] *or whether it's a telephone appointment, with a search button for the hospital and location”*  *“…; help to set a reminder; and an option of a recurring appointment or 'on-going' treatment option for outpatients’ appointments”* |
| ***Theme 3: Improvement of the GRAPHS screen*** |  | “*I don’t think I would change anything about the Graphs I love being able to see my progress and how my condition is going”*  *“A last year view might be helpful”*  *“…not sure if it's worth adding an 'albumin' section as for kidney patients like myself with NS it's helpful to see what my albumin levels are doing as that indicates if my condition is improving or not, the less albumin in my blood the better”* |
| ***Theme 4: Improving the ADD A READING screen*** |  | *“…maybe say that you can select more than one option on the oedema section or make it a list rather than a drop down? Until I accidentally selected another option I didn't realise I could choose more than 1. Maybe adding the intensity of swelling on a scale of 1-5 or 10 will also help gauge a difference to next times readings. Also having other metric units example, Ibs / st as well as Kg for weight”*  *“Maybe add an extra symptom notes box for things that are not oedema or temp etc”*  *“Make an option to copy the previous test result such as protein tests instead of having to fill out all of the details for the same result every day. This is mainly due to being in remission and being negative for consecutive days in a row.”*  *“A notes section may be useful but not necessary”* |
| ***Theme 5: Improving the DID YOU KNOW screen*** |  | “…a marker to show where to go, and Maybe under each section a bit of general advice for that topic  “..reassurance that the app will be updated every so often.” |
| ***Theme 6: Further comments*** |  | *“I think overall the app is great it just might need a few changes to make it a bit less of a hassle to track certain things such as filling in all the details of a reading, but this could be fixed by adding a copy of a previous reading or something similar. Also, in one of the text boxes I made a typo and tried to click on the part where I made the typo but instead of going to the place in the text it just took me out of the text box and if I wanted to fix it I had to backspace to the point where I'd made the mistake. Apart from those two points I think the app is great and will be very useful and is easy to use”*  *“Maybe a forum to keep in touch with other young people with NS”*  *“Not sure if you're allowed to do this but maybe a section on light exercise that one can do to keep fit and healthy?”* |
