# Supplementary material for: The NeST (Nephrotic Syndrome Trust) App, a novel, co-designed self-management support app for young people and young adults with Nephrotic Syndrome: a multi-method survey reporting initial app development and evaluation
Source: BMC Nephrol. 2025 Dec 15;27:52. doi: 10.1186/s12882-025-04684-1 (PMC12822111; doi:10.1186/s12882-025-04684-1)

## Additional File 2 – Word Clouds relating to the six emergent themes

**Theme 1- Improving the RENAL Screen?**

*An example word cloud display of the combined responses to Q16:*


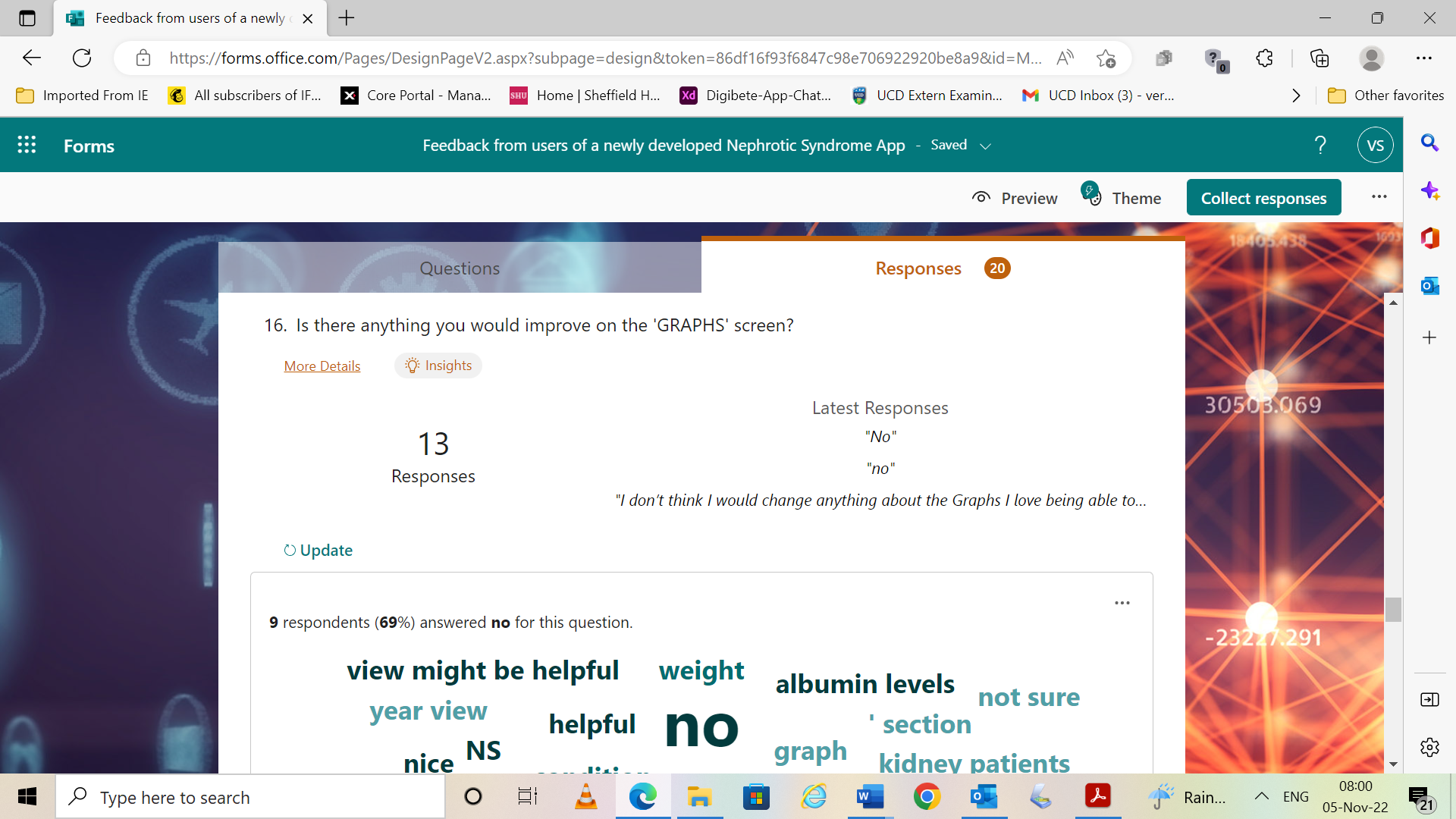


*Word cloud relating to suggested improvements to the App:*


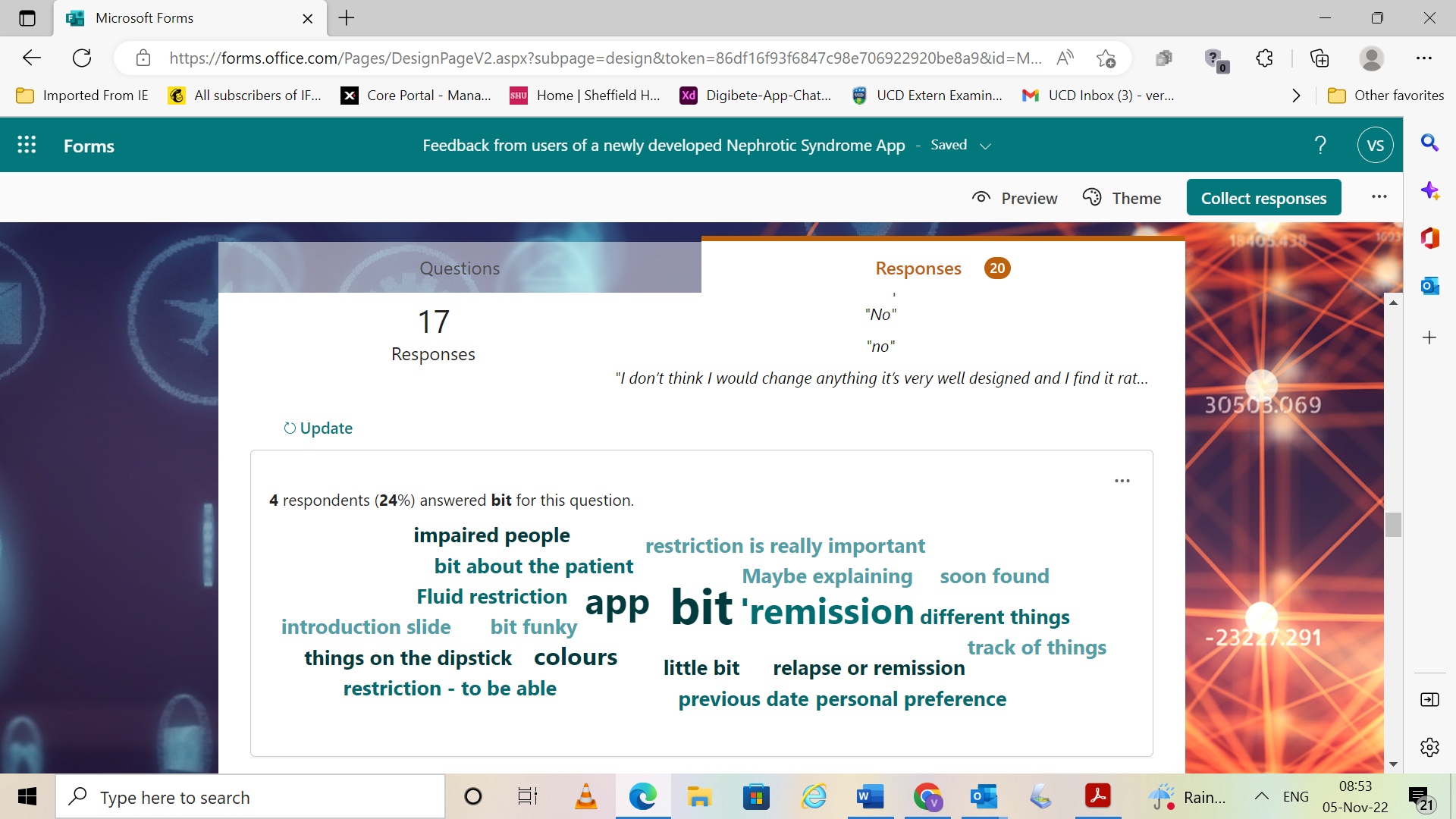


**Theme 2: Improving the ADD APPOINTMENT screen**

*Word cloud relating to suggested improvements to the ADD APPOINTMENT section of the App:*


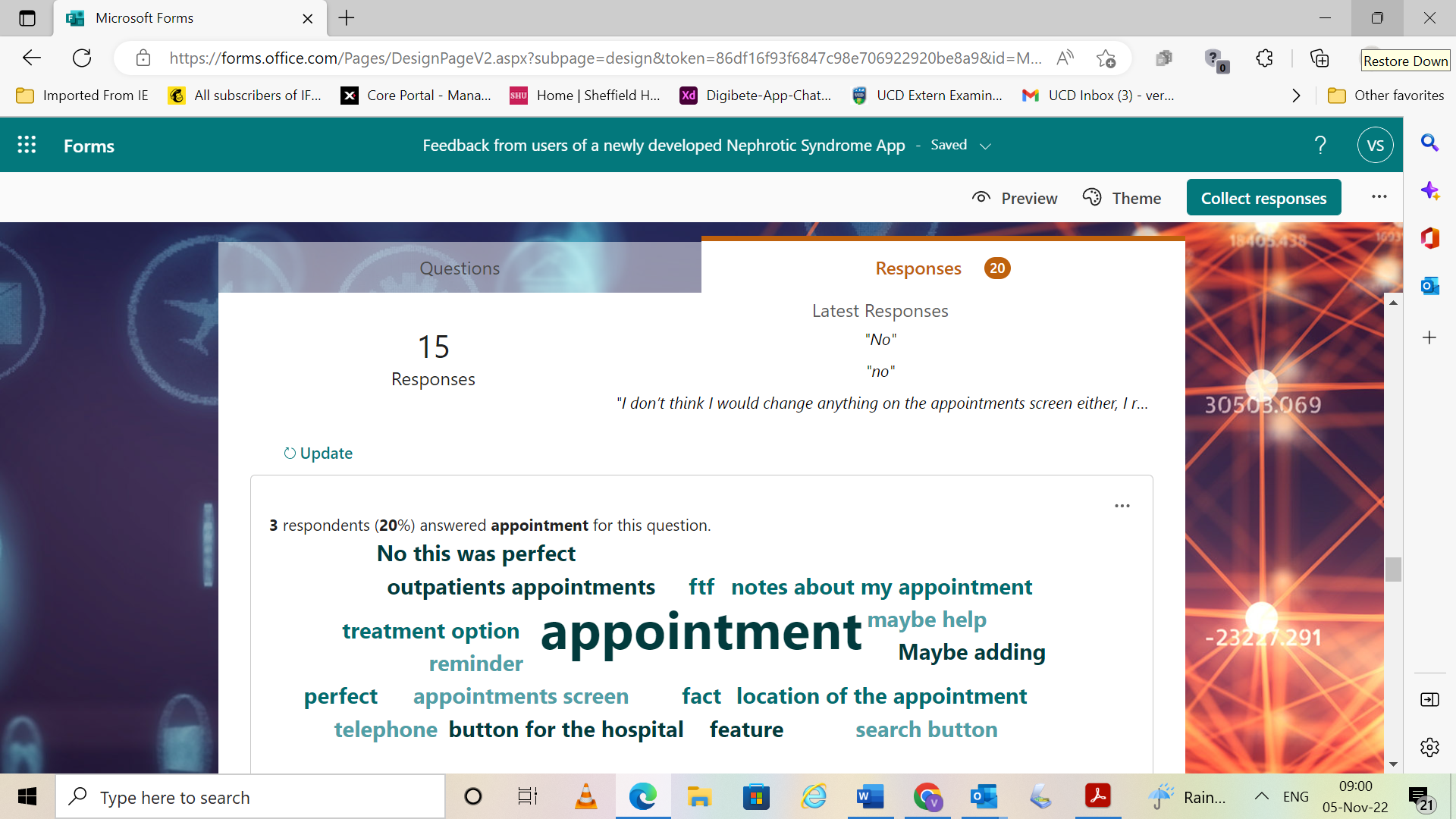


**Theme 3: Improvement of the GRAPHS screen**

*Word cloud relating to the possibility of improvements to the GRAPHS screen section of the App:*


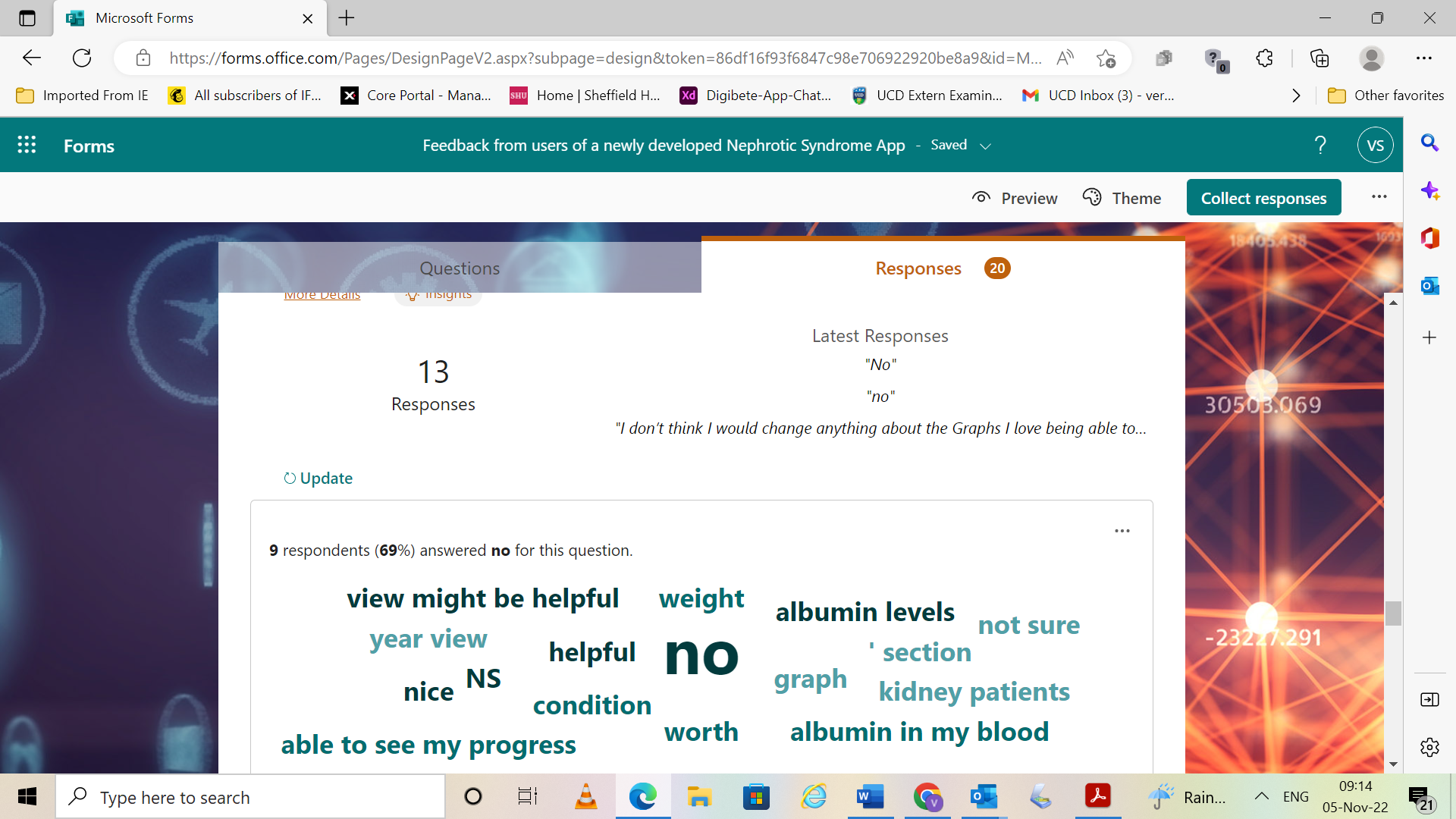


***Theme 4:* Improving the ADD A READING screen**

*Word cloud relating to suggested improvements to the* ADD A READING *screen section of the App:*


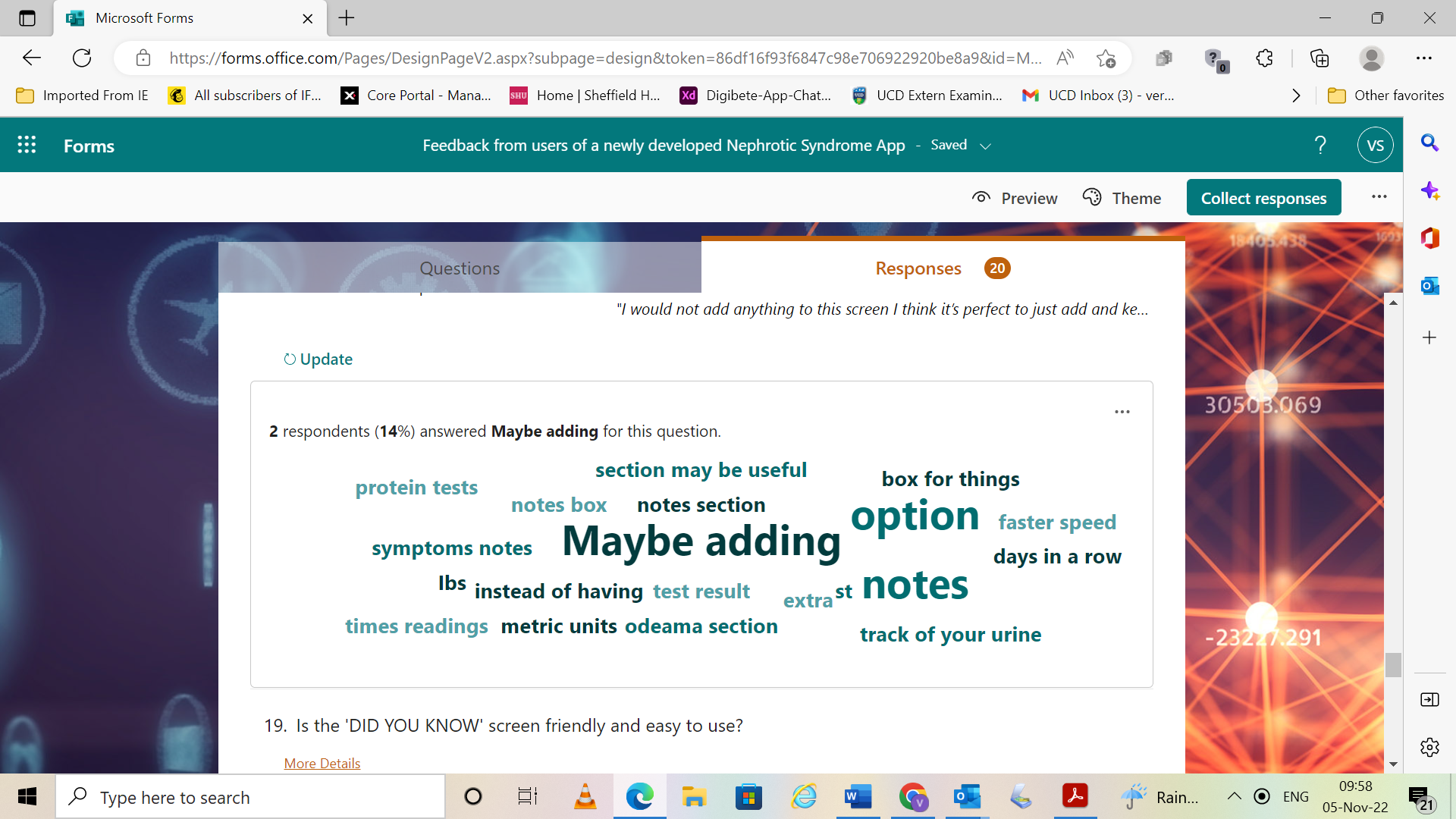


***Theme 5:* Improving the DID YOU KNOW screen**

*Word cloud relating to suggested improvements to the* DID YOU KNOW *screen section of the App:*


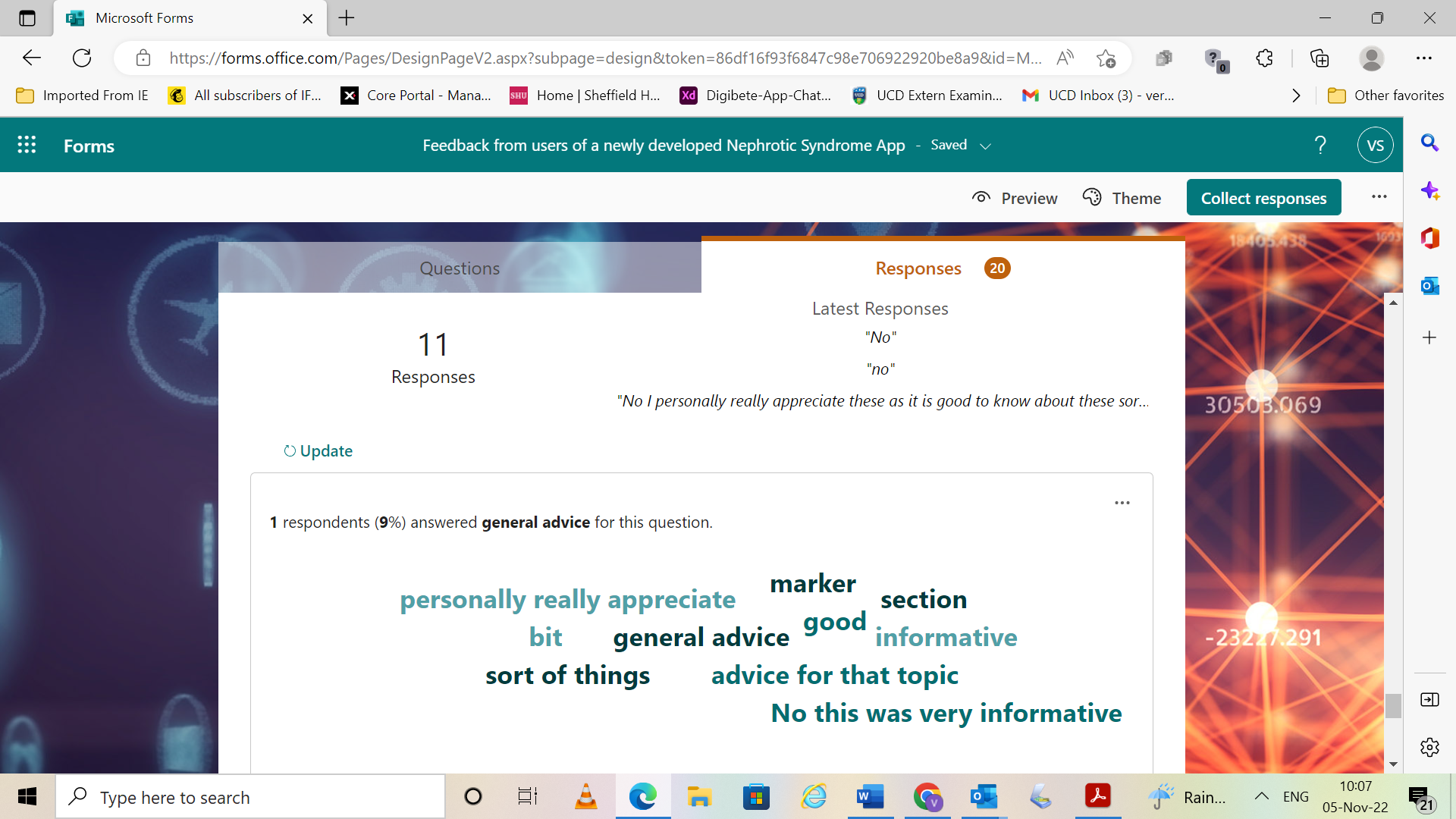


***Theme 6: Further comments***

*Word cloud relating to suggested improvements to the* FURTHER COMMENTS AND ANY PROBLEMS *screen section of the App:*


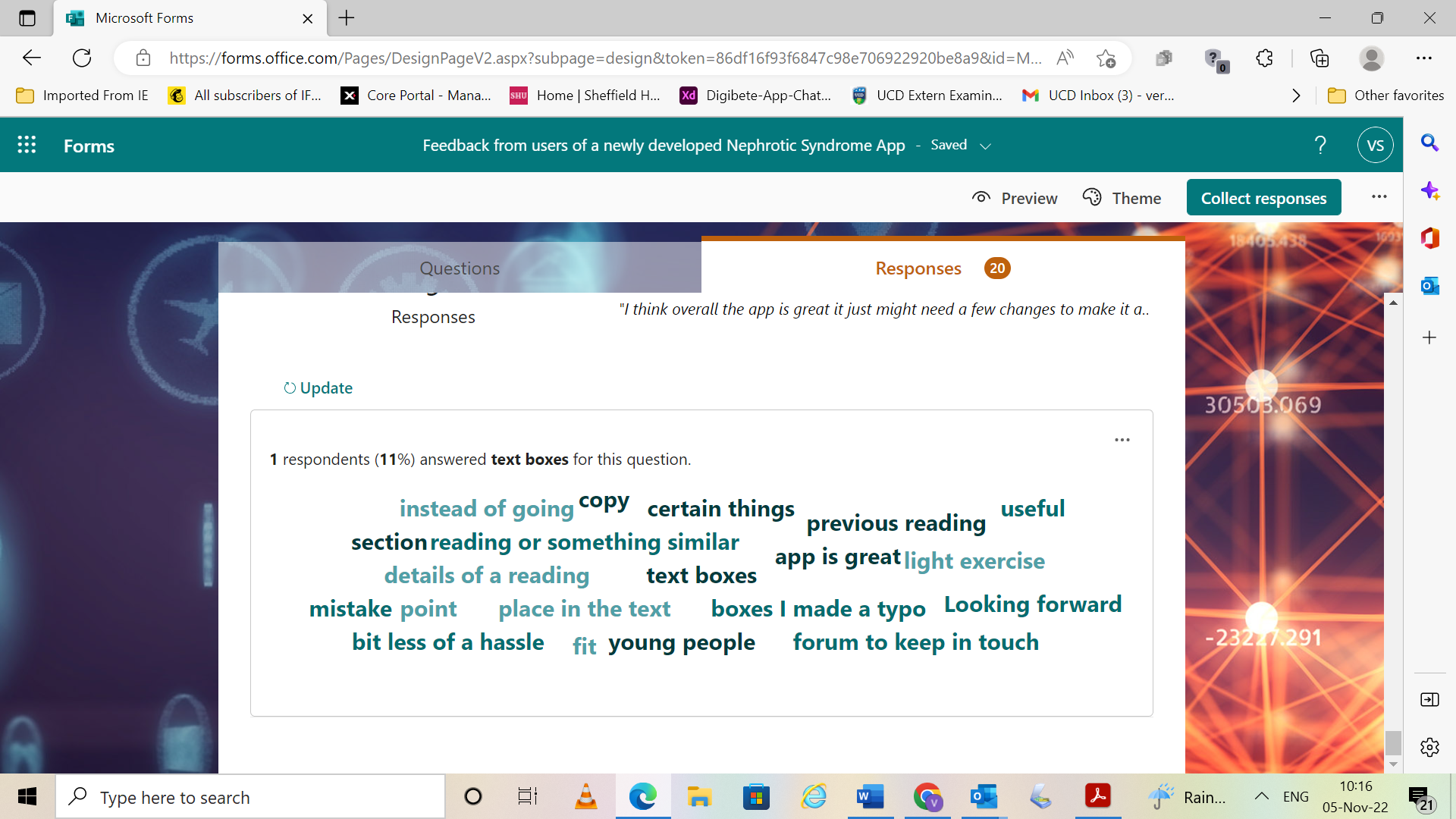

Supplement: Supplementary file 3 — Supplementary Material 3 [file 12882_2025_4684_MOESM3_ESM.docx]
