# Supplementary material for: The NeST (Nephrotic Syndrome Trust) App, a novel, co-designed self-management support app for young people and young adults with Nephrotic Syndrome: a multi-method survey reporting initial app development and evaluation
Source: BMC Nephrol. 2025 Dec 15;27:52. doi: 10.1186/s12882-025-04684-1 (PMC12822111; doi:10.1186/s12882-025-04684-1)
Supplement: Supplementary file 4 — Supplementary Material 4 [file 12882_2025_4684_MOESM4_ESM.docx]

# Appendices

## Appendix 1: Participant information sheet and invitation message

**Project title:** A survey to assess the views of young people living with Nephrotic Syndrome on a newly developed digital app

**SHORT TITLE: Surveying a new App. and Nurturing Nephrotic Futures**

*******************************************************************************

**Email message:**

Subject: Invitation to complete an anonymous questionnaire about your views on this new App.

Hello

We are writing to invite you to take part in a short survey (Up to 5 minutes) that is funded by the Wellcome charity. We attach a participant information sheet. To complete the survey please follow this link [LINK TO SURVEY] by [DATE].

Best wishes

[The research team]

**Twitter message:**

What are your views on this new App.? Here is your invitation to a survey to shape (or further develop) the app to help young people living with Nephrotic Syndrome [TINYURL]

*************************************************************************************

***Participant Information Sheet***

You are invited to take part in the above project. This is funded by the Wellcome charity and being carried out by a team of nephrotic syndrome patients, parents, doctors, and researchers in the UK, led by Mrs Wendy Cook, Director of the Nephrotic Syndrome Trust (NSTrust <https://nstrust.co.uk/> )

We are doing this project because we know that nephrotic syndrome mostly affects young children and young adults. We are developing a novel app that we hope can improve communication between patients with nephrotic syndrome, researchers, and clinicians. Nephrotic syndrome can affect people's ability to work, play, plan their lives around their treatments and to form relationships. Clinicians want to better understand how nephrotic syndrome affects patients. This new app will help patients to share their experiences of living with nephrotic syndrome in clinics and with researchers. This is an important piece of research into this rare disease.

We therefore want to ask your views on this first version of the App. by asking you to download and use the App. and then answer the short questions in this survey [ link to survey]. The survey is anonymous and takes approximately 2-5 minutes to complete. Taking part is voluntary; by completing the survey you will have agreed to take part. You do not have to answer any questions that you do not want to. Once you have completed and submitted your responses you are not able to cancel or change your answers.

We believe there are no risks to you from taking part in the survey. As with any online activity the risk of a breach is always possible. To the best of our ability your participation in this study will remain confidential, and only anonymised data will be published.

Raw data will be destroyed as soon as we have finished sharing the results with the funder, and clinicians and other researchers. In our reporting of the results no person will be identified.

The study will close on [DATE]

Thank you for considering this invitation. If you have any questions before deciding whether to take part, please contact:

Signed [The research team]

## Appendix 2: Survey questions

App Survey Questions

1. Age range
2. First language
3. Gender
4. Do you use any existing apps or devices to help manage your condition?
5. Do your parents or guardians help you manage your condition?
6. How long ago do you think you or your parent/guardian first noticed your NS symptoms?
7. Do you find this App helpful?
8. Would you use this App?
9. Is this App Easy to use?
10. I would need help to use the app
11. Is the home screen 'RENAL' friendly and easy to use?
12. Is there anything you would improve on the 'RENAL' screen?
13. Is the 'ADD APPOINTMENT' screen friendly and easy to use?
14. Is there anything you would improve about the 'ADD APPOINTMENT' screen?
15. Is the 'GRAPHS' screen friendly and easy to use?
16. Is there anything you would improve on the 'GRAPHS' screen?
17. Is the 'ADD A READING' screen friendly and easy to use?
18. Is there anything you would add to the 'ADD A READING' screen?
19. Is the 'DID YOU KNOW' screen friendly and easy to use?
20. Is there anything you would add to the 'DID YOU KNOW' screen?

Thank you for completing the questionnaire.

## Appendix 3: NeST App recommendations arising from the survey

- Regular updating on the information provided
- Addition of a section on medication,
- A process for monitoring fluid intake
- Information on interpreting the results in a urine dipstick
- Option to record how long been in remission
- Adding the location of the appointment or if it's a telephone appointment
- Adding a search button for the hospital and location
- Help to set a reminder for appointments.
- An option of a recurring appointment or 'on-going' treatment option for outpatients’ appointments
- Addition of a last year view
- Addition of an 'albumin' section
- Add more than one option on the oedema section or make it a list rather than a drop down
- Add the intensity of swelling on a scale of e.g. 1-5 or 10
- Include other metric units’ example, Ibs / st as well as Kg for weight
- Add an extra symptom notes box for things that are not oedema or temp etc.
- Make an option to copy the previous test result such as protein tests instead of having to fill out all of the details for the same result every day
- Add a notes section
- On the did You Know section include a marker to show where to go, and Maybe under each section a bit of general advice for that topic.
- To help with tracking certain things such as filling in all the details of a reading, but this could be fixed by adding a copy previous reading or something similar
- A forum to keep in touch with other young people with NS
- A section on light exercise that one can do to keep fit and healthy

# List of Appendices

Appendix 1: Survey invitation and Participant Information Sheet

Appendix 2: Survey questions

Appendix 3: Recommendations arising from the survey responses
